# Hepatoprotective Activity of *Cichorium endivia* L. Extract and Its Chemical Constituents

**DOI:** 10.3390/molecules16119049

**Published:** 2011-10-27

**Authors:** Chao-Jie Chen, An-Jun Deng, Chang Liu, Rui Shi, Hai-Lin Qin, Ai-Ping Wang

**Affiliations:** 1 New Drug Safety Evaluation Center, Institute of Materia Medica, Peking Union Medical College and Chinese Academy of Medical Sciences, No. 1 Xiannongtan Street, Beijing 100050, China; Email: chaojienchen@yahoo.com (C.-J.C.); 2 Key Laboratory of Bioactive Substances and Resources Utilization of Chinese Herbal Medicine, Institute of Materia Medica, Peking Union Medical College and Chinese Academy of Medical Sciences, No. 1 Xiannongtan Street, Beijing 100050, China; Email: denganjun@imm.ac.cn (A.-J.D.)

**Keywords:** *Cichorium endivia* L., hepatoprotective activity, oxidaitive damage, 2-furanmethanol-(5'→11)-1,3-cyclopentadiene-[5,4-c]-1*H*-cinnoline, kaempferol

## Abstract

The objective of the present study was to investigate the *in vitro* and *in vivo* hepatoprotective properties of *Cichorium endivia* L. extract (CEE), and to identify its chemical constituents. CEE significantly blocked the oxidative stress and cytotoxicity induced by *tert-*butyl hydroperoxide (*t*-BHP) in HepG2 cells. Meanwhile, oral administration of CEE to mice before the treatment of *t*-BHP exhibited a markedly protective effect by lowering serum levels of ALT and AST, inhibiting the changes in liver biochemistry including MDA, SOD, GSH and GST, as well as ameliorating the liver injuries according to the histopathological observations. According to the acute oral toxicity test, the LD_50_ of CEE was greater than 5,000 mg/kg, which demonstrates that the CEE can be considered practically non-toxic. Phytochemical analysis of CEE showed the presence of five compounds identified as 2-furanmethanol-(5'→11)-1,3-cyclopentadiene-[5,4-c]-1*H*-cinnoline, which is a new cinnoline derivative derived from a natural source but not synthesis, 2-phenylethyl-*β*-D-glucopyranoside, kaempferol-3-*O*-β-D-glucoside, kaempferol, and adenosine. In the ORAC assay, CEE and its constituents kaempferol and kaempferol-3-*O*-β-D-glucoside had considerable antioxidant potency. Taken together, CEE protects hepatic tissue from oxidative damage *in vitro* and *in vivo*, potentially due to its phenolic substances, and does not cause acute oral toxicity, which suggests that CEE may be a valid and safe remedy to cure liver disease.

## 1. Introduction

Due to the limited prevention and treatment options, liver diseases are considered to be one of the most serious health problems in the World [1]. Exposure of the liver to the free radicals derived from some xenobiotics and drugs leads to oxidative stress, which is recognized to be an important factor responsible for liver injury or be involved in the pathogenesis of liver disorders [2,3]. Therefore, studies on scavenging free radicals or reactive oxygen species (ROS) as well as reducing oxidative stress, and thereby avoiding hepatotoxicity, have received much attention [4,5,6].

*Cichorium endivia* L. (Compositae), either cooked or eaten raw in salads, is a favorite cultivated vegetable around the World. The popularity of *C. endivia* is attributed to its healthy properties, which are mainly due to its high levels of antioxidant compounds [7]. In China, there are three species in the genus *Cichorium*: *Cichorium intybus* L., *C. glandulosum* Boiss. Et Huet and *C. endivia* L. Except *C. endivia*, the another two *Cichorium* species are considered to be folk medicines used for the treatment of liver diseases [8], and their hepatoprotective effects related to their antioxidant capacity was demonstrated in previous studies [9,10,11]. Nevertheless, *C. endivia* is also capable of scavenging free radicals, as well as protecting the microsome membrane of rat hepatocytes and *Staphylococcus aureus* cultures from oxidative injury [12,13]. In addition, it shares some biological active constituents such as sesquiterpenes and phenolic compounds with *C. intybus* [14,15,16]. Whereas there are a relatively large number of studies concerning the pharmacological and biological action of *C. intybus*, the bioactivity of *C. endivia* has been largely neglected. In our previous study, we found that a 60% ethanol eluate extract from *C. endivia* named CEE showed considerable antioxidant potency *in vitro* [17]. According to these results, we put forth a hypothesis of hepatoprotective activity of CEE associated with its antioxidant capacity, and present in the present study the hepatoprotective activities and phytochemistry of the extract.

## 2. Results and Discussion

### 2.1. *In Vivo* Hepatoprotective Activity of CEE

**Figure 1:**
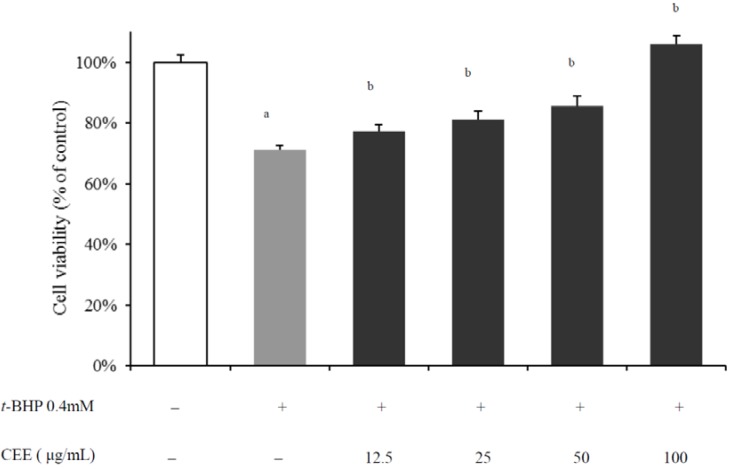
Effect of CEE on cytotoxicity of *t*-BHP-induced HepG2 cells (N = 6). HepG2 cells were treated with CEE (12.5, 25, 50 or 200 μg/mL) in the presence of *t*-BHP (0.4 mM) for 3 h, and cell viability was determined by MTT assay. ^a^*p* < 0.01, compared with control; ^b^*p* < 0.01, compared with *t*-BHP-treated cells. CEE: *Cichorium endivia* L. extract; *t*-BHP: *tert*-butyl hydroperoxide.

**Figure 2:**
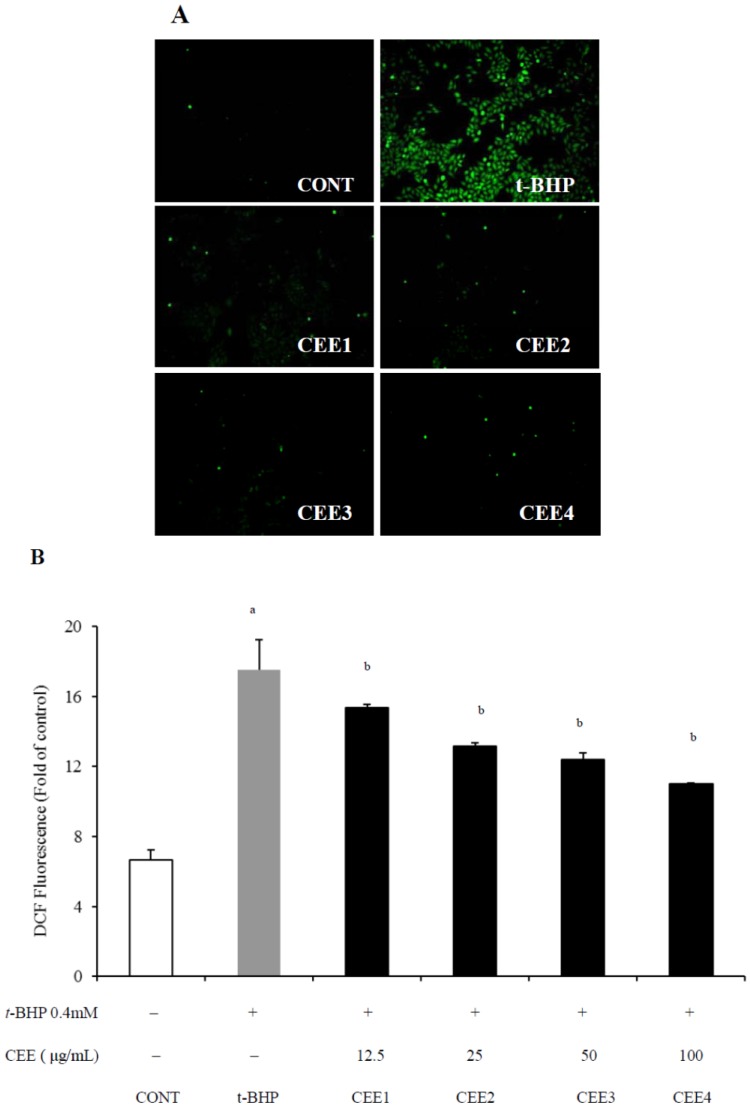
Effect of CEE on ROS Production in *t*-BHP treated HepG2 cells (N = 3). Cells were co-treated with *t*-BHP (0.4 mM) and CEE (12.5, 25, 50, or 200 μg/mL) for 1 h. The fluorescent probe DCFH-DA (50 μM) was added 30 min before monitoring the level of intracellular ROS with (**A**) a fluorescence microscope and (**B**) fluorometer. DCFH-DA staining, magnification 100×; ^a^*p* < 0.01, compared with control group. ^b^*p* < 0.01, compared with *t*-BHP-treated cells. **CONT**: Non-treated control cells, *t*-**BHP**: *t*-BHP treated cells; **CEE1,2,3** and **4** represent 12.5, 25, 50, and 200 μg/mL of CEE, respectively. CEE: *Cichorium endivia* L. extracts; DCFH-DA: 2′,7′-dichlorofluorescin diacetate; *t*-BHP: *tert*-butyl hydroperoxide.

To confirm the antioxidant capacity as well as the hypothesis of hepatoprotective activtiy on CEE, HepG2 cells were co-treated with *tert-*butyl hydroperoxide (*t*-BHP) and different concentrations of CEE for 3 h (for the cell viability assay) or 1 h (for intracellular ROS production measurements), respectively. As shown in Figure 1, the viability of HepG2 cells treated by 0.4 mM *t*-BHP alone decreased to 71.35 ± 1.13% of the normal control group. CEE prevented *t*-BHP-induced cell death (*p* < 0.01) and the cytotoxicity-inhibitory activity was dependent on the concentration of CEE. In the meantime, an increase in ROS production was observed over time in the presence of 0.4 mM *t*-BHP compared to unstressed controls (*p* < 0.01). With addition of CEE, the fluorescence intensities were significantly (*p* < 0.01) and concentration-dependently diminished compared to *t*-BHP only-treated cells (Figure 2). The results mentioned above suggest that CEE reduced *t*-BHP-induced cell death associated with its effects on the suppression of intracellular ROS production. Thus, the antioxidant bioactivity of CEE on hepatocyte, as an efficient inhibitor of intracellular ROS production as well as a good protector against hepatic damage, is demonstrated.

### 2.2. *In Vivo* Hepatoprotective Activity of CEE

In addition to the biological activity in HepG2 cells, CEE was expected to display hepatoprotective activity *in vivo*. To confirm this, the *t*-BHP-induced acute liver injury mice model was used. Many studies have pointed out that *t*-BHP, as a well-known pro-oxidant, causes leakage of alanine aminotransferase (ALT) and aspartate aminotransferase (AST), formation of malondialdehyde (MDA), and reduction of level of glutathione (GSH) and activities of antioxidant enzymes in rodents [18,19,20,21,22]. These evidences suggest that liver injury induced by *t*-BHP in animals can serve as an *in vivo* model for screening the hepatoprotective activities of drugs.

**Figure 3:**
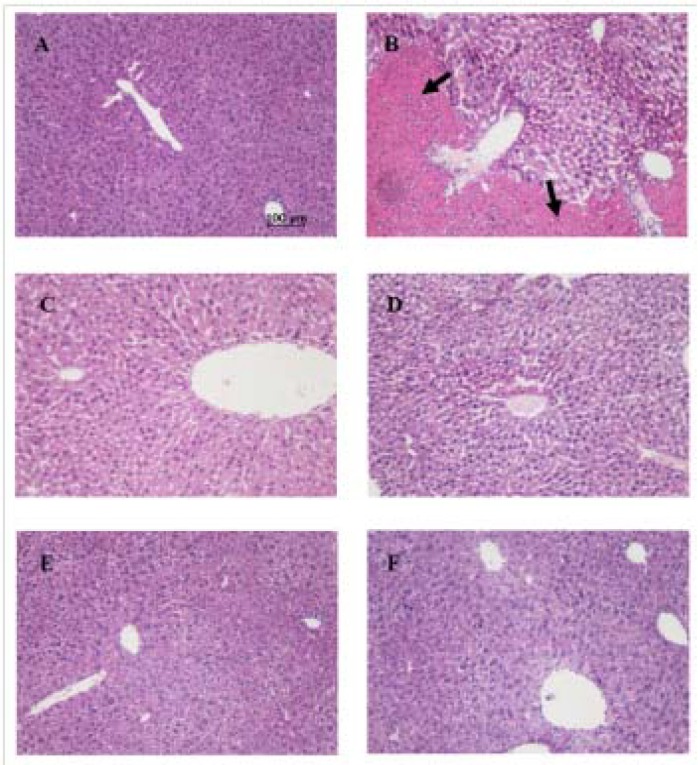
Effect of CEE on *t*-BHP-induced liver damage in mice. The mice were pretreated with CEE (200, 400 or 800 mg/kg, i.g.) and DDB (150 mg/kg, i.g.) once daily for 8 consecutive days. Vehicle control mice were given normal saline. Four hours after the final treatment, the mice were treated with *t-*BHP (0.2 mmol/kg i.p.). Mice were sacrificed 24 h after *t*-BHP administration. (**A**) Vehicle control group; (**B**) Animals treated with *t*-BHP (0.2 mmol/kg), showed acidophilic degeneration with areas of patchy necrosis in parenchyma (arrow). Animals treated with (**C**) DDB, and (**D**) 200, (**E**) 400 or (**F**) 800 mg/kg CEE, respectively, and then with *t*-BHP showed normal morphology; hematoxylin and eosin staining; magnification 100×. CEE: *Cichorium endivia* L. extracts; DDB: diphenyl dimethyl bicarboxylate; *t*-BHP: *tert*-butyl hydroperoxide.

A single dose of *t*-BHP given to mice by injection i.p. after 24 h caused elevations of serum ALT and AST, increased levels of MDA, led to depletion of GSH, and reduced activities of antioxidant enzymes superoxide dismutase (SOD) and glutathione S-transferase (GST) in the liver compared with the vehicle control group (Table 1, *p* < 0.05 or 0.01). Pretreatment of CEE significantly attenuated the effect of *t*-BHP (*p* < 0.05 or 0.01), and its hepatoprotective effect at high dosage (800 mg/kg body weight) was comparable to the reference agent diphenyldimethyl bicarboxylate (DDB, 150 mg/kg body weight, i.g.), a result further indicating that CEE has the potential to reduce the hepatotoxicity induced by *t*-BHP. Histological examination showed that treatment of mice with *t*-BHP alone led to acidophilic degeneration with areas of patchy necrosis in parenchyma (Figure 3B), but pretreatment with CEE and DDB inhibited the pathological change of liver injury (Figure 3C–F).

Consistent with the evidences mentioned above, our results showed that acute administration of *t*-BHP produced a marked elevation of the serum levels of ALT and AST in treated animals when compared with that of the vehicle control group. Pretreatment with CEE significantly reduced the elevated levels of the enzymes. Decreased serum levels of ALT and AST by CEE is an indication of stabilization of plasma membrane as well as repair of hepatic tissue damage caused by chemical pro-oxidant. The above changes can be considered as an expression of the functional improvement of hepatocytes, which may be caused by an accelerated regeneration of parenchyma cells [23].

**Table 1:** Effect of CEE on t-BHP-induced hepatotoxicity in mice *.

Group	Serum ALT(Karmen unit)	Serum AST(Karmen unit)	MDA(μmol/g protein)	SOD(U/mg protein)	GSH(μmol/g protein)	GST(U/mg protein)
Vehicle control	11.88 ± 4.31	24.75 ± 4.56	0.81 ± 0.20	290.20 ± 10.88	8.25 ± 1.06	32.90 ± 1.36
*t-*BHP treatment	198.70 ± 30.72 ^b^	118.03 ± 11.58 ^b^	2.28 ± 0.29 ^b^	222.30 ± 17.41 ^b^	2.46 ± 0.32 ^b^	27.81 ± 2.78 ^a^
150 mg/kg DDB	33.16 ± 14.15 ^d^	41.13 ± 14.45 ^d^	1.07 ± 0.27 ^d^	262.69 ± 15.36 ^d^	6.10 ± 0.39 ^d^	36.32 ± 4.14 ^d^
200 mg/kg CEE	144.28 ± 17.17 ^c^	84.52 ± 10.94 ^d^	1.52 ± 0.16 ^d^	246.18 ± 5.86 ^c^	3.08 ± 0.57 ^c^	32.42 ± 5.03
400 mg/kg CEE	75.85 ± 14.83 ^d^	51.67 ± 15.22 ^d^	1.22 ± 0.11 ^d^	253.09 ± 6.50 ^c^	4.05 ± 0.24 ^d^	35.64 ± 2.87 ^d^
800 mg/kg CEE	33.10 ± 9.71 ^d^	38.76 ± 9.50 ^d^	1.03 ± 0.07 ^d^	271.47 ± 13.38 ^d^	4.79 ± 0.31 ^d^	36.14 ± 1.81 ^d^

*The mice were pretreated with CEE (200, 400 or 800 mg/kg, i.g.) once daily for 8 consecutive days. Control mice were given normal saline. Four hour after the final treatment, the mice were treated with *t-*BHP (0.2 mmol/kg i.p.). Hepatotoxixity was determined 24 h later by quantifying the serum activities of ALT and AST, as well as hepatic lipid peroxidation, SOD, GSH and GST. Values are expressed as the mean ± S.D. (N = 8). Means within the same column bearing different superscript letters are significantly different (*p* < 0.05) as determined by analysis of variance by Dunnett's test. ^a^*p* < 0.05, ^b^*p* < 0.01, compared with vehicle control group, ^c^*p* < 0.05, ^d^*p* < 0.01, compared with *t*-BHP-treated group. ALT: alanine aminotransferase; AST: aspartate aminotransferase; CEE: *Cichorium endivia* L. extracts; DDB: diphenyldimethyl bicarboxylate; GSH: glutathione; GST: glutathione S-transferase; i.g.: intragastrically; i.p.: intraperitoneally; MDA: malondialdehyde; SOD: superoxide dismutase; *t-*BHP: *tert*-butyl hydroperoxide.

The body has an effective antioxidant defense system against free radicals and ROS induced damage, in which the endogenous enzymatic and non-enzymatic antioxidants such as GSH, SOD and GST play an important role [24,25]. GSH is as an essential intracellular reducing substance for the maintenance of thiol groups on intracellular proteins and antioxidant molecules in living organisms [26]. Perturbation of GSH status in a biological system has been reported to lead to serious consequences [27]. SOD, GST and other antioxidant enzymes constitute a mutually supportive team of defense against ROS. SOD is a metalloproteinase to detoxify superoxide anions as an efficient dismutative mechanism and is the first enzyme involved in the antioxidant defense [28]. GTS plays a vital role in liver by eliminating toxic compounds by conjugating them with GSH [29]. However, once the balance between ROS production and antioxidant defenses is lost, oxidative stress will consequently occur, which through a series of biological events deregulates the cellular functions leading to various pathological conditions [27]. In the present study, elevated level of MDA in *t*-BHP-treated mice indicates excessive formation of free radicals and activation of lipid peroxidation system resulting in hepatic damage. MDA produced as byproducts of lipid peroxidation that occurs in hydrophobic core of bio-membranes [30]. The significant decline in the concentration of MDA in the mice’s liver tissue treated with both *t*-BHP and CEE indicates anti-lipid peroxidative effect of *C. endivia.* Meanwhile, pretreatment of CEE effectively blocked the *t*-BHP reduced abnormal changes in the level of GSH, and the activity of SOD and GST in mice liver reveals that CEE has a potent antioxidant property towards chemical-induced hepatic injury.

### 2.3. Acute Oral Toxicity of CEE in Mice

Acute oral toxicity of CEE for mice of 10 males and 10 females was assessed following a single dose administered by gavage at a dose of 5,000 mg/kg body weight. Mortality, clinical signs and body weights of the mice were measured for 14 days following the administration of CEE. All animals gained weight and did not show any abnormal signs within the trial period. The mean body weight of female mice increased from 18.11 ± 1.26 g to 24.29 ± 2.21 g during the period, and that of males increased from 19.23 ± 1.68 g to 33.53 ± 1.54 g. After sacrifice on the 14th day, macroscopic and gross pathology observations conducted at the necropsy examination revealed no visible lesions in any animals. Thus, no evidence of acute toxicity of CEE in mice was found. The oral LD_50_ values for female and male mice must be greater than 5,000 mg/kg body weight. According to the literature [31], substances that present LD_50_ higher than 5,000 mg/kg by oral route can be considered practically non-toxic. As such, in the present study, significant adverse health effects following a therapy with CEE would not be expected, but this must be confirmed by further toxicological studies.

### 2.4. Structural Determination of Major Compounds from CEE

**Figure 4:**
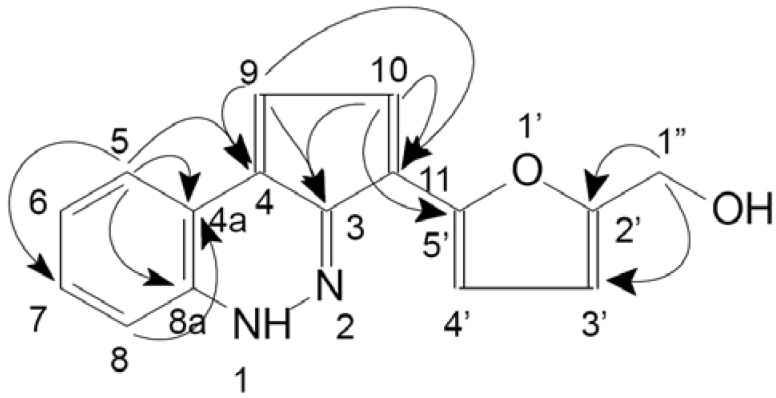
Chemical structure and key HMBC correlations (H→C) of compound **1** (2-furanmethanol-(5'→11)-1,3-cyclopentadiene-[5,4-c]-1H-cinnoline).

Five compounds **1–5** have been isolated so far as the main components of CEE. Compound 1 gave the molecular formula C_16_H_12_N_2_O_2_ by HRESIMS ([M+H]^+^*m/z* 265.0975; required 265.0972), which was confirmed by its NMR spectra. Besides a singlet due to a methyleneoxy group at *δ_H_* 4.70 (2H, s), two AB-type spin systems belonged to olefinic protons at *δ_H_* 8.22 (1H, d, *J* = 5.1 Hz) and 7.92 (1H, d, *J* = 5.1 Hz), and 7.16 (1H, d, *J* = 3.0 Hz) and 6.52 (1H, d, *J* = 3.0 Hz). An ABCD-type spin system that showed the characteristics of the *o*-substituted benzene ring at *δ_H_* 8.10 (1H, d, *J* = 8.1 Hz), 7.63 (1H, d, *J* = 8.1 Hz), 7.51 (1H, t, *J* = 7.5 Hz), 7.21 (1H, t, *J* = 7.5 Hz) were determined from the signals in the ^1^H-NMR spectrum of compound **1**. The ^13^C-NMR spectrum of **1** showed 16 carbon signals, which were ascribed to one methylene, eight methines, and seven quaternary carbons with the analysis of HSQC spectrum. This result was consistent with that of the ^1^H-NMR spectrum. The identification of **1** as a new compound, 2-furanmethanol-(5'→11)-1,3-cyclopentadiene-[5,4-c]-1*H*-cinnoline, was unambiguously demisntrated by the result of the HMBC spectrum, which showed long-range correlations of *δ_H_* 4.70 with *δ_C_* 157.2 (C-2') and 110.96 (C-3'), *δ_H_* 7.92 with *δ_C_* 134.2 (C-11), 154.2 (C-5'), 132.1 (C-4), and 132.4 (C-3); *δ_H_* 8.22 with *δ_C_* 132.1 (C-4), 122.1 (C-4a), and 132.4 (C-3), *δ_H_* 8.10 with *δ_C_* 122.1 (C-4a), 132.1 (C-4), and 143.0 (C-8a). Other key long-range correlations in the HMBC spectrum are shown in Figure 4.

Compounds **2**, **3**, **4** and **5** were identified as 2-phenylethyl-*β*-D-glucopyranoside (**2**) [32]; kaempferol-3-*O*-β-D-glucoside (**3**) [33]; kaempferol (**4**) [34,35] and adenosine (**5**) [36]; and their structures were identified by comparing spectroscopic data (^1^H- and ^13^C-NMR, MS) with literature values.

### 2.5. Antioxidant Activity of the CEE and Its Compounds

**Figure 5:**
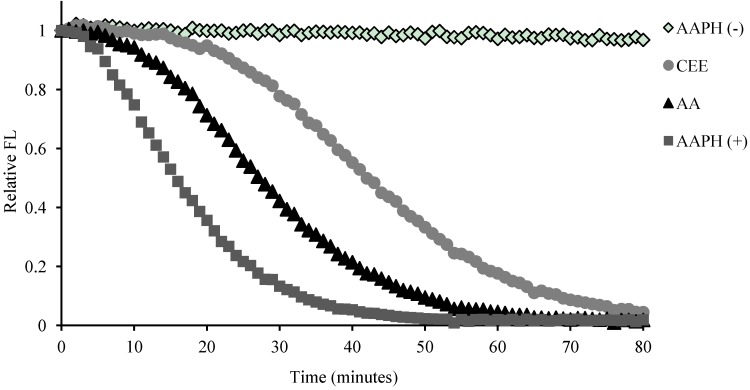
Fluorescence (FL) decay curve during oxygen radical absorbance capacity (ORAC) assay in the presence of CEE (60% ethanol eluate of *C. endivia*, 2 μg/mL), and AA (ascorbic acid, 2 μg/mL). AAPH (−): the blank which treated without 2,2′-Azobis (2-methylpropionamidine) dihydrochloride (AAPH) and any other samples; AAPH (+): the blank treated with AAPH and without any other samples.

The result of ORAC assay (Figure 5) exhibited that the antioxidant activity of CEE (ORAC values = 7675.54 μmol TE/g) was approximately two times stronger than ascorbic acid (ORAC values = 3529.32 μmol TE/g or 0.62 ± 0.01 μmol TE), which suggests that CEE had considerable antioxidant potency potentially associated with its hepatoprotective activity. Besides, the most potent antioxidant in these compounds was kaempferol followed by kaempferol-3-*O*-β-D-glucoside (Table 2). 

**Table 2:** Antioxidant activity of the constituents from CEE in ORAC assay *.

Compound	1	2	3	4	5	AA
ORAC value (μmol TE)	0.85 ± 0.01	a	10.20 ± 0.13	15.30 ± 0.19	1.12 ± 0.03	0.62 ± 0.01

* The constituents from *C. endivia* include 2-Furanmethanol-(5'→11)-1,3-cyclopentadiene-[5,4-c]-1H-cinnolin (**1**), 2-phenylethyl-β-D-glucopyranoside (**2**), kaempferol-3-O-β-D-glucoside (**3**), kaempferol (**4**) and adenosine (**5**). AA: ascorbic acid, a reference agent. The determinations on the presence of various samples were done in triplicate for three independent measurements, and the values are expressed as the mean ± S.D. (N = 3); ^a^ No activity observed.

Adenosine and 2-furanmethanol-(5'→11)-1,3-cyclopentadiene-[5,4-c]-1*H*-cinnoline had moderate antioxidant activity. No activity of 2-phenylethyl-*β*-D-glucopyranoside was detected, in agreement with the previous literature [37]. Kaemferol, one of flavonoids found in plant-derived foods that are intrinsic components of human diets, is considered to be a potent antioxidant. The antioxidant characteristic of kaemferol is attributed to its possession of B-ring hydroxyl configuration, a 2-3 double bond with a 4-oxo function and 3-hydroxyl free group, which are responsible for electron delocation between the A- and B- rings, stabilizing the flavonoid radical and preventing redox cycling after hydrogen donation [38]. The 3-hydroxyl group on kaempferol is considered to be the most important feature responsible for its antioxidant activity [39,40]. Our results confirm the importance of the 3-hydroxyl in that the antioxidant activity of kaempferol-3-*O*-β-D-glucoside was less potent than kaempferol in the ORAC assay. The literature demonstrates that the glycosylation of kaempferol at C-3 moderates the antioxidant activity, due to 3-*O*-glycosylation interfering with the coplanarity of the B-ring with the rest of the flavonoid and the ability to delocalize electrons [38,41,42]. Besides kaempferol-3-*O*-β-D-glucoside, there are another two kaempferol conjugates in *C. endivia*: kaempferol-3-*O*-β-D-glucuronide and kaempferol-3-*O*-(6-*O*-malonyl)-glucoside [43], but neither was isolated from CEE in the present study. Anyway, according to the present results, kaempferol and kaempferol-3-*O*-β-D-glucoside were considered to be the major antioxidant active compounds in CEE thus far. Futher, Kim *et al*. reported that kaempferol had significantly hepatoprotective activity [44], but kaempferol-3-*O*-β-D-glucoside failed to show it [45]. According to these, kaempferol more than its conjugate(s) may play an important role as the major constituent responsible for hepatoprotective activity in CEE.

Adenosine is an endogenous and ubiquitous nucleotide, which is a breakdown product of adenosine triphosphate and has a wide range of physiological functions in organisms. Adenosine has been reported as an antioxidant due to its purine base possessing nitrogen and oxygen atoms, which may quench radicals and chelate certain metal ions [46], however in this study the antioxidant activity of adenosine was not significant among the compounds from *C. endivia*.

Interestingly, the isolated new compound 2-furanmethanol-(5'→11)-1,3-cyclopentadiene-[5,4-c]-1*H*-cinnoline is being reported for thefirst time as a cinnoline derivative found in *C. endivia* and even in Nature. Cinnoline is a toxic nitrogenous organic base and has antibacterial activity against *Escherichia coli* [47]. None of its derivatives have been found in Nature [48]. Synthetic cinnoline compounds are of the interest due to their broad spectrum of pharmacological activities, such as antimicrobial activities [49], anti-inflammatory properties [50], antitumor activity [51], and so on. Although 2-furanmethanol-(5'→11)-1,3-cyclopentadiene-[5,4-c]-1*H*-cinnoline was not as potent as other compounds from *C. endivia* as an antioxidant in this study, the other potential bioactivity has intrigued us into studying it further in the future.

## 3. Experimental

### 3.1. General

2,2′-Azobis(2-methylpropionamidine) dihydrochloride (AAPH), ascorbic acid (AA), 2′,7′-dichloro-fluorescin diacetate (DCFH-DA), ethylenediaminetetraacetic acid (EDTA), 3-(4,5-dimethylthiazol-2-yl)-2,5-diphenyl-2*H*-tetrazolium bromide (MTT) and Trolox were purchased from Sigma-Aldrich (St. Louis, MO, USA). Fluorescein (FL) were purchased from Sinopharm Chemical Reagent Beijing Co., Ltd (Beijing, China). *tert*-Butylhydroperoxide (*t*-BHP) was purchased from Sinopharm Chemical Reagent Beijing Co. Ltd. (Beijing, China). 1,1,3,3-Tetramethoxypropane (TEP) was purchased from J&K Scientific Ltd. (Beijing, China). Kits for a Bio-Rad protein assay, ALT, AST, SOD, GSH and GST were purchased from Nanjing Jiancheng Bioengineering Institute (Nanjing, China). Diphenyldimethyl bicarboxylate (DDB) was purchased from Beijing Union Pharm (Beijing, China). Other chemicals and solvents were of analytical grade. ^1^H- and ^13^C-NMR spectra were recorded on a Varian Mercury-400 spectrometer. Chemical shifts (δ) were given in ppm using tetramethylsilane (TMS) as internal standard (δ 0.00). ESI/MS were measured on an Agilent 1100 series LC-MSD-Trap-SL spectrometer. Preparative HPLC was carried out on a Shimadzu LC-6AD, equipped with a SPD-10A detector. A reversed-phase C18 column (YMC-Pack ODS-A Ф 20 × 250 mm, 10 µm) was employed. Column chromatography (CC) was performed with silica gel (200–300 mesh, Qingdao Marine Chemical Group Co., Qingdao, China) and Sephadex LH-20 (Pharmacia Biotech AB, Uppsala, Sweden). TLC was carried out with glass plate precoated silica gel G. Spots were visualized under UV light or by spraying with 10% H_2_SO_4_ in 95% EtOH followed by heating.

### 3.2. CEE Preparation

*C. endivia* was purchased from a local market in Beijing on July 2009, and was authenticated by Professor Ma Lin (Institute of Material Medica, Chinese Academy of Medical Sciences and Peking Union Medical College). A voucher specimen (No. 1082) was deposited at the New Drug Safety Evaluation Center, Institute of Materia Medica, Peking Union Medical College and Chinese Academy of Medical Sciences, Beijing, China. The air-dried whole plant of *C. endivia* (5.8 kg) was extracted three times under conditions of reflux with 95% ethanol (EtOH, 80 L, 2 h; 70 L, 1 h; 68 L, 1 h). The combined ethanol extracts were suspended in 70% aq EtOH (3.5 L). The resulting suspension was defatted by petroleum ether (2 L, 1.5 L, 1.5 L, 1.5 L), extracted with ethyl acetate (EtOAc, 1.5 L, 1.2 L, 1.2 L), applied to a Daion HP-20 column (10 × 60 cm), and eluted with a H_2_O-EtOH series (100:0, 14 L; 40:60, 15 L; 5:95, 15 L). The 60% EtOH fraction was evaporated under vacuum to yield a black residue (40 g, CEE), which was dissolved in dimethylsulfoxide (DMSO) for *in vitro* experiments, and suspended in normal saline for the *in vivo* hepatoprotective test and acute oral toxicity study, respectively.

### 3.3. Cell Culture

HepG2 cells kindly donated by Professor Li Yan (Institute of Materia Medica, Peking Union Medical College and Chinese Academy of Medical Sciences) were cultured in DMEM medium containing 10% fetal calf serum (FCS) under a 5% CO_2_ atmosphere at 37 °C.

### 3.4. Cell Viability Assay

For cell viability assay [18], HepG2 cells were dispensed into 96 well plates at the concentration of 1 × 10^4^ cells per well. After 24 h incubation, cells were co-treated with *t*-BHP (0.4 mM) and various concentrations of CEE (dissolved in 0.5% DMSO) for 3 h. The cells were added with MTT (0.5 mg/mL) and incubated for another 4 h, and then the cell medium was replaced by 200 μL DMSO. Absorbance at 570 nm was determined with a microplate reader (Molecular Devices Spectra MAX 190, Sunnyvale, CA, USA) and used for the measurement of the proportion of surviving cells. Control cells were treated with 0.5% DMSO and various concentrations of CEE, respectively, and they had no noticeable effect on the assay system.

### 3.5. Measurement of Intracellular ROS Level

To measure intracellular ROS production [22], HepG2 cells were planted in 6-well plates (2 × 10^6^ cells per well) for 24 h incubation, and then were exposed to *t*-BHP (0.4 mM) and various concentrations of CEE for 1 h. The fluorescent probe 2′,7′-dichlorofluorescin diacetate (DCFH-DA, 50 μM) was added 30 min before monitoring the level of intracellular ROS with a fluorescence microscope (Olympus, IX-70, Nagano, Japan) and a fluorometer at an excitation of 485 nm and an emission of 530 nm using a fluorescence microplate reader (Molecular Devices Spectra Max Gemini XS, Sunnyvale, CA, USA). No noticeable effect of 0.5% DMSO and various concentrations of CEE on intracellular ROS production had been observed.

### 3.6. Animals

ICR mice of either sex (weighing 18–20 g) were purchased from Vital River Laboratory Animal Technology Co. Ltd. (Beijing, China). The Administrative Committee on Animal Research in the Institute of Materia Medica, Peking Union Medical College and Chinese Academy of Medical Sciences approved all the protocols for animal experiments. Also, all the animal experiments were performed in compliance with the Guiding Principles for the Care and Use of Laboratory Animals, Peking Union Medical College, China. The animals were provided standard rodent chow/feed and water *ad libitum*.

### 3.7. Acute Liver Injury Induced by t-BHP in Mice

Male ICR mice were randomly divided into six groups (8 mice/group). To study the protective effect against the *t*-BHP-induced hepatotoxicity, CEE (200, 400 or 800 mg/kg body weight) and positive reference DDB was administrated intragastrically (i.g.) to the animals for 8 consecutive days. On day 8, the animals were intraperitoneally (i.p.) given injection of *t*-BHP (0.2 mmol/kg body weight) except for the vehicle control group, and 24 h later the mice were sacrificed by decapitation under anesthesia and the blood samples were collected for the assays of ALT and AST. The livers were excised from the animals and assayed for the level of malondialdehyde (MDA), SOD, GSH and GST, and pathological histology was performed according to the procedures described below.

#### 3.7.1. Hepatotoxicity Assessment

The hepatic enzymes ALT and AST were used as the biochemical indicators for the acute liver injury. The serum ALT and AST activities were determined by commercial kits (Nanjing Jiancheng Bioengineering Institute, Nanjing, China).

#### 3.7.2. Lipid Peroxidation Assay

Lipid peroxidation assay was performed as described in [52], with small modifications. Briefly, after being weighed, the livers were minced into small pieces, and rinsed twice with ice-cold homogenization buffer composed of 250 mM sucrose, 50 mM Tris-HCl, pH 7.4, 1 mM EDTA, and then homogenized in nine volumes of ice-cold homogenization buffer employed by a Teflon pestle. The homogenates were centrifuged at 1,000 g for 10 min, and the supernatants were centrifuged at 12,000 g for 30 min again. The final supernatant protein contents were determined by a commercial Bio-Rad protein assay (Nanjing Jiancheng Bioengineering Institute, Nanjing, China). Lipid peroxidation product, MDA, was assayed according to an improved thiobarbituric acid fluorometric method at 515 nm ex/550 nm em using TEP as the standard [53].

#### 3.7.3. Antioxidant Enzyme Activity Assay

The liver homogenate supernatants collected as previously described were used for the SOD, GSH and GST assay followed by the commercial kits (Nanjing Jiancheng Bioengineering Institute).

#### 3.7.4. Pathological Histology

The liver tissues were removed from the animals and immediately fixed in 10% formalin. Subsequent processing included dehydrating in increasing ethanol solutions (50–100%), clearing in xylene and embedding in paraffin. Sections (4–5 μm) were prepared and then stained with hematoxylin/eosin dye for photomicroscopic observations.

### 3.8. Acute oral Toxicity Test

A single-dose oral study was conducted in ICR mice to evaluate the potential toxicity of high exposure to CEE. After overnight fasting (12 h), groups of 10 male and 10 female mice were administrated 5,000 mg/kg body weight by oral gavage. Each animal was provided with water and food *ad libitum* 4 h after the treatment. Signs or symptoms of possible toxicity and for mortality were observed every hour for the first 6 h and every day for 14 days. On the 14th day, all mice were sacrificed under anesthesia and subjected to necropsies [54,55].

### 3.9. Oxygen Radical Absorbance Capacity (ORAC) Assay

The antioxidant activity was determined following a procedure similar to that previously described [56]. The reaction was carried out in potassium phosphate buffer (75 mM, pH 7.4). FL and AAPH were dissolved in the phosphate buffer in a certain concentration. The samples of extracts, compounds, and reference standards were dissolved in DMSO, and then diluted with phosphate buffer (0.1% DMSO, v/v). The samples (20 μL) in different concentrations (0−2 μg/mL of extracts, and 0–10 μmol/L of constituents) mixed in 96 black well microplates (Costar, USA) with FL (100 μL, 122.4 nM) and maintained at 37 °C for 5 min. Oxidation reaction was started after adding AAPH (80 μL, 47.5 mM) to each well. The FL fluorescence was excited at 485 nm, and the fluorescence emission was detected at 538 nm. The decay of FL fluorescence was monitored every 1 min at 37 °C until the fluorescence of the last reading had declined to less than 5% of the first reading using a fluorescence microplate reader (Molecular Devices Spectra Max Gemini XS, USA). The microplate was shaken prior to each reading. Final fluorescence measurements were expressed relative to the initial reading. Results were calculated based on differences in areas under the FL decay curve between the blank and a sample. They were all expressed as trolox equivalents (μmol/g weight of extracts or μmol/μmol of compounds). Ascorbic acid was used as a positive control.

### 3.10. Statistical Analysis

Statistical analysis was performed using SPSS (version 11.0; SPSS, Chicago, IL, USA).The data were expressed as means ± SD, and significant differences were determined by One-way analysis of variance (ANOVA) followed with Duncan’s multiple range tests. A *p*-value of less than 0.05 was considered statistically significant.

### 3.11. Isolation and Identification of CEE

In order to isolate the main compounds from CEE and determine their chemical structures, the 60% ethanol fraction was re-dissolved in *n*-butanol and washed with aq. 5% NaHCO_3_ and H_2_O (2 × 1000 mL), respectively. Evaporation of *n*-butanol under reduced pressure gave 5.5 g of brown green residue, which was dissolved in methanol and purified by preparative RP-HPLC eluted with 60%, 80%, and 100% methanol in water to provide 13 fractions according to the HPLC profile. Fraction 2 was repurified by preparative RP-HPLC eluted with 40% methanol in water to give compound **1** (70 mg). Fraction 4 was repurified by preparative RP-HPLC eluted with 40% methanol in water to give compound **2** (24 mg) and **3** (43 mg). Fraction 10 was repurified by preparative RP-HPLC eluted with 80% methanol in water to give compound **4** (31 mg). Fraction 13 was repurified by preparative RP-HPLC eluted with 75% methanol in water to give compound **5** (13 mg).

### 3.12. Characterization of 2-furanmethanol-(5'→11)-1,3-cyclopentadiene-[5,4-c]-1H-cinnoline (**1**)

White amorphous powder; ESI-MS: *m/z* 265 [M+H]^+^, 287 [M+Na]^+^; HRESIMS: *m/z* 287.0794 [M+Na]^+^ (calcd for C_16_H_12_N_2_O_2_Na, 287.0791), 265.0975 [M+H]^+^ (calcd for C_16_H_13_N_2_O_2_, 265.0792); ^1^H-NMR (300 MHz, CD_3_OD) *δ*: 8.21 (1H, d, *J* = 5.1 Hz, H-9), 8.10 (1H, d, *J* = 8.1 Hz, H-5), 7.92 (1H, d, *J* = 5.1 Hz, H-10), 7.63 (1H, d, *J* = 8.1 Hz, H-8), 7.51 (1H, t, *J* = 7.5 Hz, H-7), 7.21 (1H, d, *J* = 7.5 Hz, H-6), 7.16 (1H, d, *J* = 3.0 Hz, H-4'), 6.52 (1H, d, *J* = 3.0 Hz, H-3'), 4.70 (2H, s, H_2_-1"); ^13^C-NMR (100 MHz, CD_3_OD) *δ*: 157.2 (C-2'), 154.2 (C-5'), 143.0 (C-8a), 138.5 (C-9), 134.2 (C-11), 132.4 (C-3), 132.1 (C-4), 130.0 (C-7), 122.5 (C-5), 122.1 (C-4a), 121.2 (C-6), 114.9 (C-10), 113.2 (C-8), 111.0 (C-3'), 110.9 (C-4'), 57.6 (C-1").

## 4. Conclusions

Our data demonstrated that CEE possessed a powerful hepatoprotective capacity and was practically non-toxic. Phytochemical analysis suggests that this capacity may be attributed to the presence of phenolic substances, especially kaemferol. Noticeably, one new compound isolated from *C. endivia*, 2-furanmethanol-(5'→11)-1,3-cyclopentadiene-[5,4-c]-1*H*-cinnoline, is a kind of cinnoline derivative that have not been found in Nature before. Taken together, the results suggest that CEE could be beneficial for health due to its antioxidant capacity, and might potentially cure liver diseases.
